# Supplementary material for: Harnessing Gene Expression Profiles for the Identification of Ex Vivo Drug Response Genes in Pediatric Acute Myeloid Leukemia
Source: Cancers (Basel). 2020 May 15;12(5):1247. doi: 10.3390/cancers12051247 (PMC7281398; doi:10.3390/cancers12051247)

Supplementary Material

Harnessing Gene Expression Profiles for the Identification of Ex Vivo Drug Response Genes in Pediatric Acute Myeloid Leukemia

DGJ Cucchi *et al.* 2020.

Supplementary methods

Mononuclear Cell Enrichment using Ficoll Pague gradient centrifugation procedure.

After bone marrow aspiration, bone marrow cells were filtered through a 70 uM cell strainer and were diluted 1:1 with Phosphate Buffered Saline (Thermo Scientific, Waltham, US) + 0.1% Human Serum Albumin (20% HSA, Sanquin, Amsterdam, The Netherlands). Cell concentrations were maximized at 20 million cells per ml Ficoll (1.077 g/ml Amersham Biosciences, Freiburg Germany). In a 50 ml tube, the diluted bone marrow sample was added very slowly on top of 15 ml Ficoll separation medium. Samples were centrifuged for 22 minutes at 900g at room temperature. After centrifugation, the interphase was carefully collected and transferred to a new 50 ml tube. The tube was complemented with PBS 0.1% HSA to 50 ml and mixed gently. Then the tubes were centrifuged for 10 minutes at 570g, after which the supernatant was discarded. Cell yield was determined and blast percentage was assessed by morphology using cytospins stained with May-Grünwald-Giems (Merck, Darmstadt, Germany).

Cytospins

The mononuclear cell suspension was set to a concentration of 0.5 x 10e6/ml and washed once. Glass slides were pre-wetted. Then, 50 µl of cell suspension was added to the cytospin chambers, followed by centrifugation at 680rpm for 7 minutes. Slides were air-dried for several minutes before staining. May-Grunwald/Giemsa staining was performed by fixing in 100% methanol for 3 minutes, then staining in May-Grunwald for 3 minutes. Slides were rinsed with tap water and subsequently stained with Giemsa for 15 minutes. After rinsing, slides were dried before microscopic evaluation.

Culture medium

Culture medium consisted of RPMI, 20% Fetal Calf Serum, 200 µg/ml Gentamycine, 2 mM glutamine, 5 µg/ml insulin, 5 µg/ml transferrin and 5 ng/ml sodiumselenite.

Leukemic cell purification

In case of low (< 75%) leukemic blasts, leukemic cell purification was performed as previously described [20] using magnetic polystyrene beads (Dynabeads M-450, Dynal, Norway) to remove excess lymphocytes using CD3. Co-expression of CD3 on blasts was evaluated before starting the procedure. Direct purification was performed by washing CD3-

coated magnetic beads twice in culture medium and incubating the beads with the mononuclear cell suspension in a 10:1 bead:cell ratio for 30 minutes at 4 ºC with continuous gentle mixing. After incubation, the tube containing beads + cells was placed in a magnetic field and the non-bound cells were removed by careful pipetting.

Ex Vivo Drug Response

Ex vivo drug response was assessed immediately after leukemic cell enrichment and thus these assays were performed on fresh AML cells. Only samples with >80% blasts were used. *Ex vivo* cytotoxicity of the deoxynucleoside analogues 1-β-D-arabinfuranosylcytosine (Ara C, Cytosar; Pharmacia &Upjohn, Woerden, The Netherlands) and 2-chlorodeoxyadenosine (2-CdA, Leustatin, Ortho Biotech, USA), the anthracycline daunorubicin (DNR, Cerubidine, Rhône-Poulenc, France) and the topoisomerase II inhibitor etoposide phosphate (VP16, etoposide-TEVA; TEVA-Pharma, Netherlands) was determined after 96 hour drug exposure using MTT. In 96-well round-bottom microplates (Greiner Bio-one, Alphen a/d Rijn, The Netherlands), six concentrations of each drug were used in the following ranges: Ara C (0.04 – 41 μM); 2-CdA (0.001 - 140 μM); DNR (0.004 – 4 μM), and VP16 (0.09 – 3.4 μM). In designated wells, 10 μl drug dilution (10x) was added. Then, 90 μl cell suspension was added to reach a total volume of 100 μl per well, with a total of 0.08 – 0.12 x 10^6 cells/well, depending on availability. Cells without any drug added were included as controls to six wells and four wells containing culture medium only were used as blanks. The outer wells were filled with 100ul ddH_20 to minimize evaporation. The plates were incubated for 96 hours at 37°C in a humidified atmosphere containing 5% CO2, after which 10 μl of 3-[4,5-349 dimethylthiazol-2-yl]-2,5 diphenyl tetrazoliumbromide (MTT; 5 mg/ml, Sigma Aldrich, Zwijndrecht, The Netherlands) was added. After six hours of additional incubation at similar conditions, formazan crystals (indicating metabolically viable cells) were dissolved using acidified isopropanol (0.04 N-HCl-isopropyl alcohol) and the optical density (OD) was measured spectophotometrically at 562 and 720 nm. Importantly, only high quality data were used with stringent criteria, since 25 % of fresh AML samples cannot be used in *ex vivo* assays due to limited viability or low (<80%) blast percentage, and 20% of MTTs are not evaluable due to low number of remaining blasts at day 4. Evaluable results were obtained when a minimum of 70% leukemic blast cells was present at day 4 in control wells and when the control OD was > 0.05. Dose response curves were obtained and drug responses were summarized using the LC_{50} value, the drug concentration achieving 50% lethality of the leukemic cells. When the LC_{50} value was not reached (in 4/121 samples), the highest concentration evaluated was used as LC_{50}.

Correlation of LC50 values to evaluate cross-resistance

To investigate cross-resistance, LC_{50} values towards the four chemotherapeutic drugs were correlated using Spearman's Rank Correlation. A p-value of 0.05 was considered as

statistically significant. Four samples (#4721 from the discovery cohort, VI, V28 and V37 from the validation cohort) were excluded from these analyses, as these samples did not reach 50% viability when tested using the MTT assay for one or two drugs evaluated (**Supplementary Table 1).**

Supplementary Figures

Supplementary Figure S1: Complete comparisons of LC_{50} values within genetic subtypes based on recurrent molecular mutations in pediatric AML. No statistical differences between LC_{50} values in wild-type and mutant samples were observed (Student's t-test, $p > 0.1$).

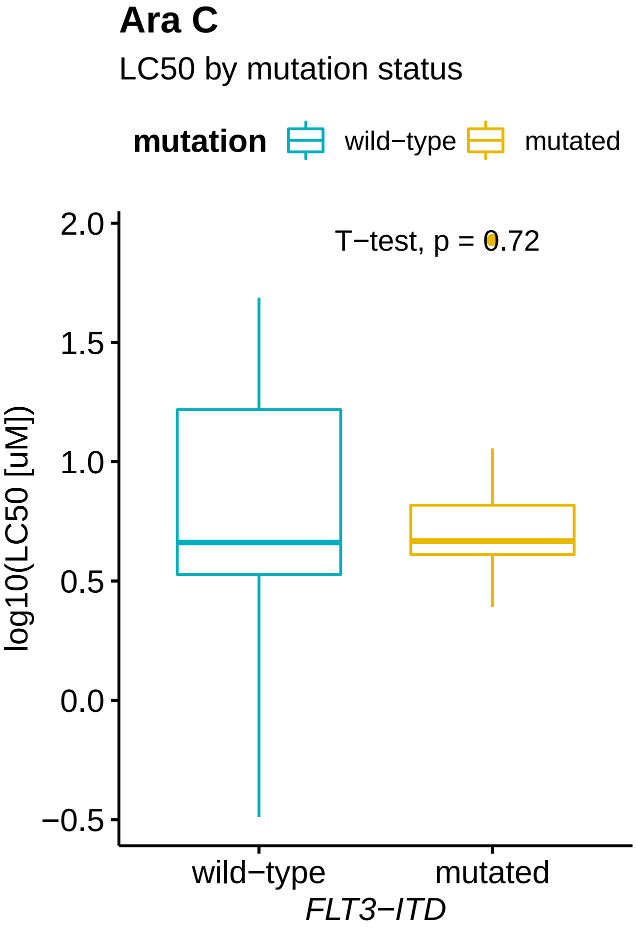
Ara C
LC50 by mutation status
mutation
wild−type
mutated
T−test, p = 0.72
log10(LC50 [uM])
2.0
1.5
1.0
0.5
0.0
−0.5
wild−type
mutated
FLT3−ITD

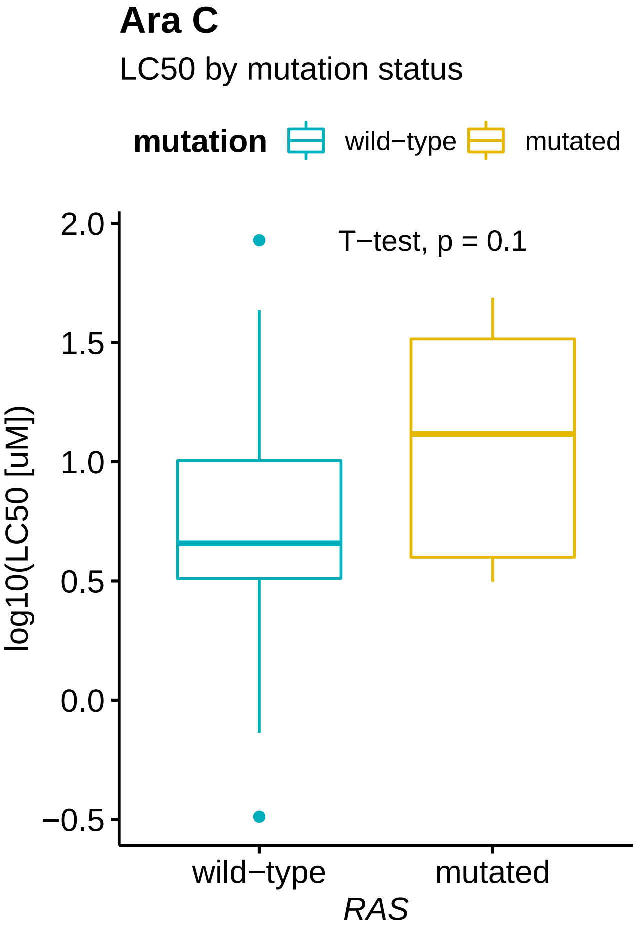
Ara C
LC50 by mutation status
mutation
wild−type
mutated
T−test, p = 0.1
log10(LC50 [uM])
2.0
1.5
1.0
0.5
0.0
−0.5
wild−type
mutated
RAS

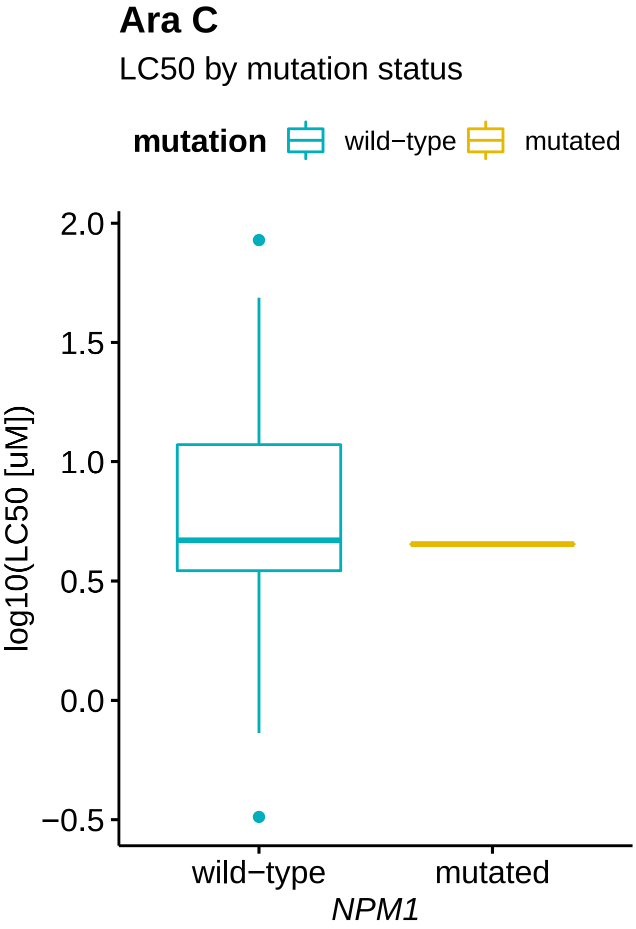
Ara C
LC50 by mutation status
mutation
wild−type
mutated
2.0
1.5
1.0
0.5
0.0
−0.5
log10(LC50 [uM])
wild−type
mutated
NPM1

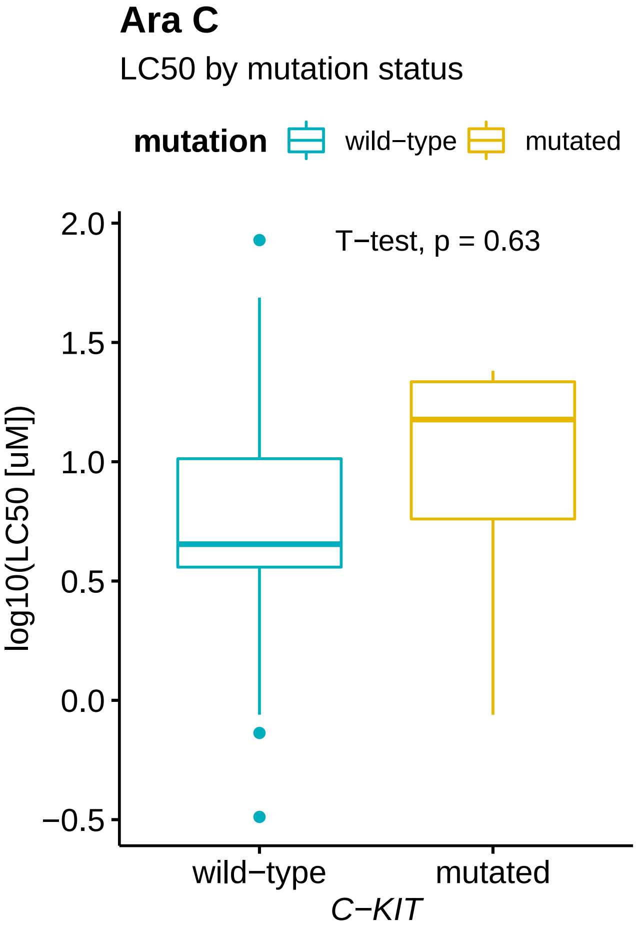
Ara C
LC50 by mutation status
mutation
wild−type
mutated
T−test, p = 0.63
2.0
1.5
1.0
0.5
0.0
−0.5
log10(LC50 [uM])
wild−type
mutated
C−KIT

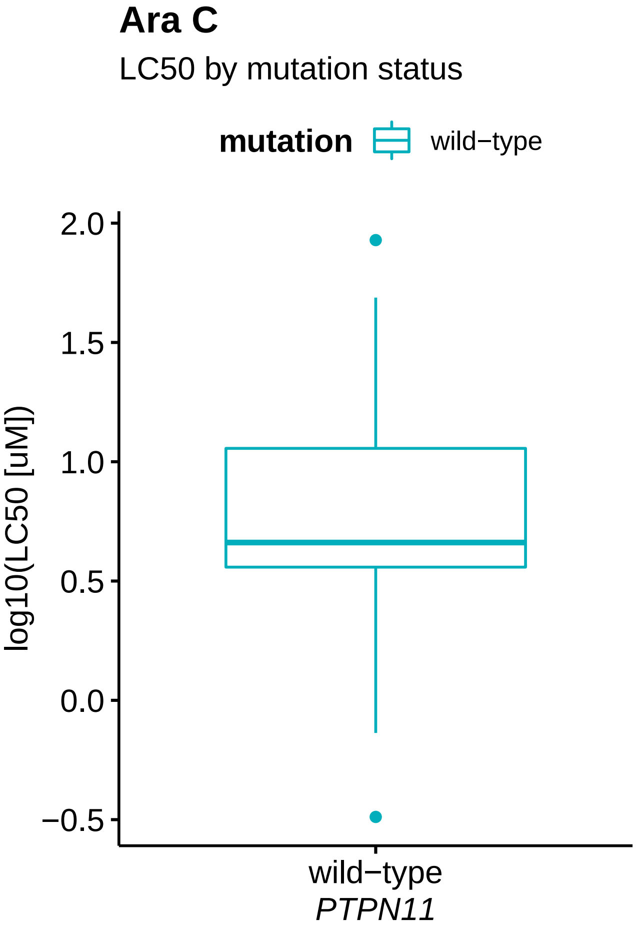
Ara C
LC50 by mutation status
mutation
wild−type
2.0
1.5
1.0
0.5
0.0
−0.5
log10(LC50 [uM])
wild−type
PTPN11

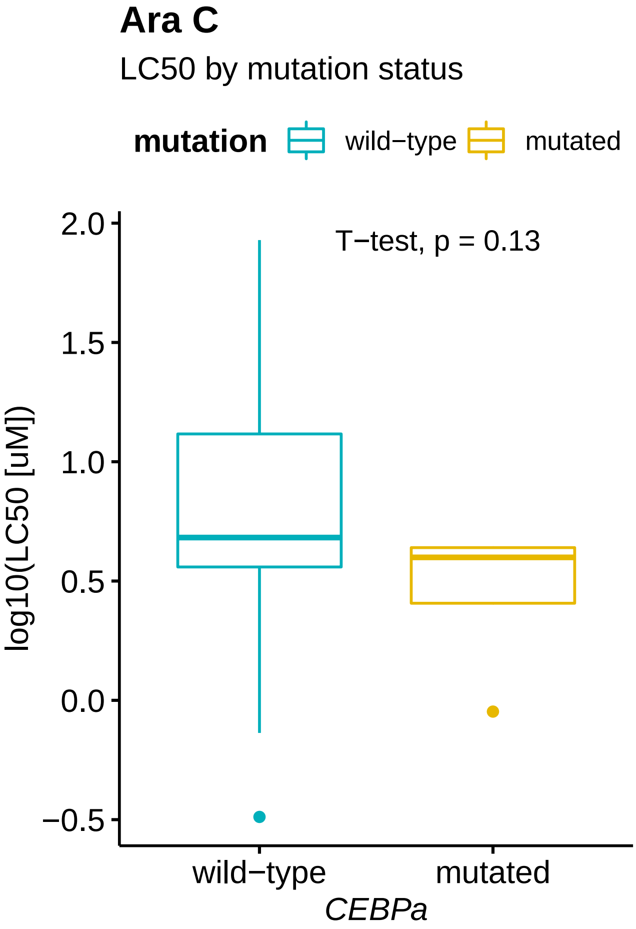
Ara C
LC50 by mutation status
mutation
wild−type
mutated
T−test, p = 0.13
2.0
1.5
1.0
0.5
0.0
−0.5
log10(LC50 [uM])
wild−type
mutated
CEBPa

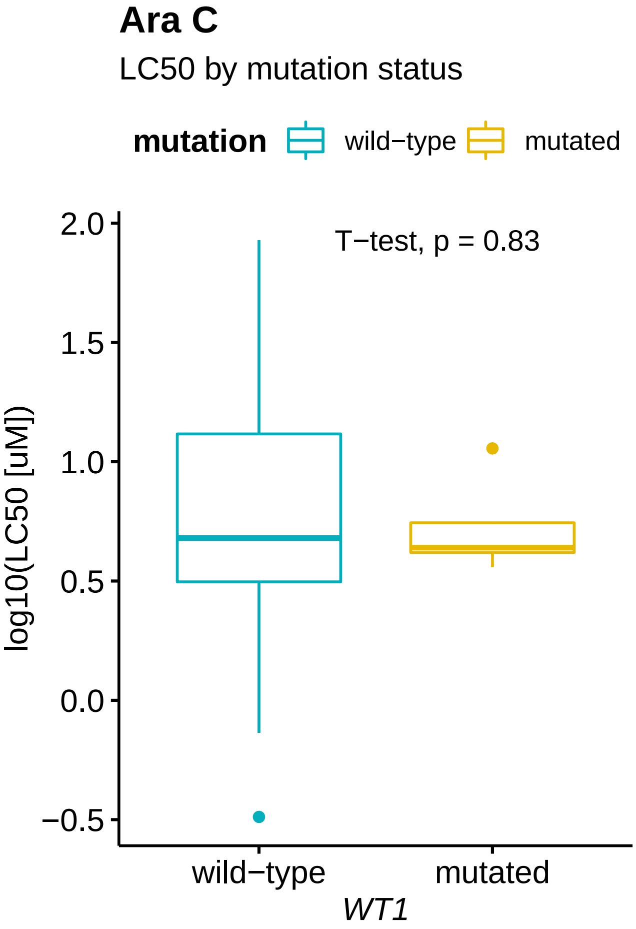
Ara C
LC50 by mutation status
mutation
wild−type
mutated
T−test, p = 0.83
log10(LC50 [uM])
2.0
1.5
1.0
0.5
0.0
−0.5
wild−type
mutated
WT1

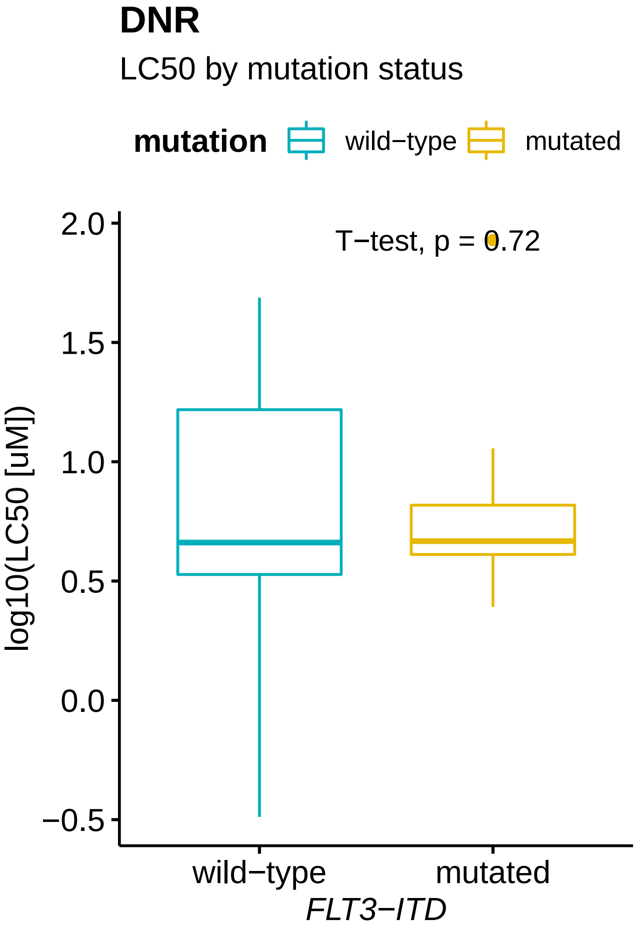
DNR
LC50 by mutation status
mutation
wild−type
mutated
T−test, p = 0.72
log10(LC50 [uM])
2.0
1.5
1.0
0.5
0.0
−0.5
wild−type
mutated
FLT3−ITD

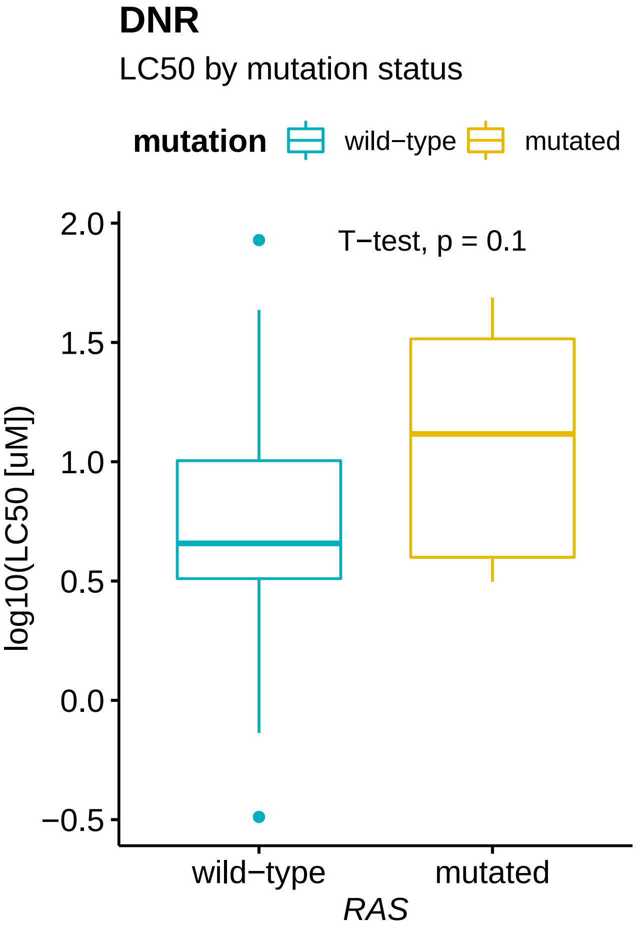
DNR
LC50 by mutation status
mutation
wild−type
mutated
T−test, p = 0.1
2.0
1.5
1.0
0.5
0.0
−0.5
log10(LC50 [uM])
wild−type
mutated
RAS

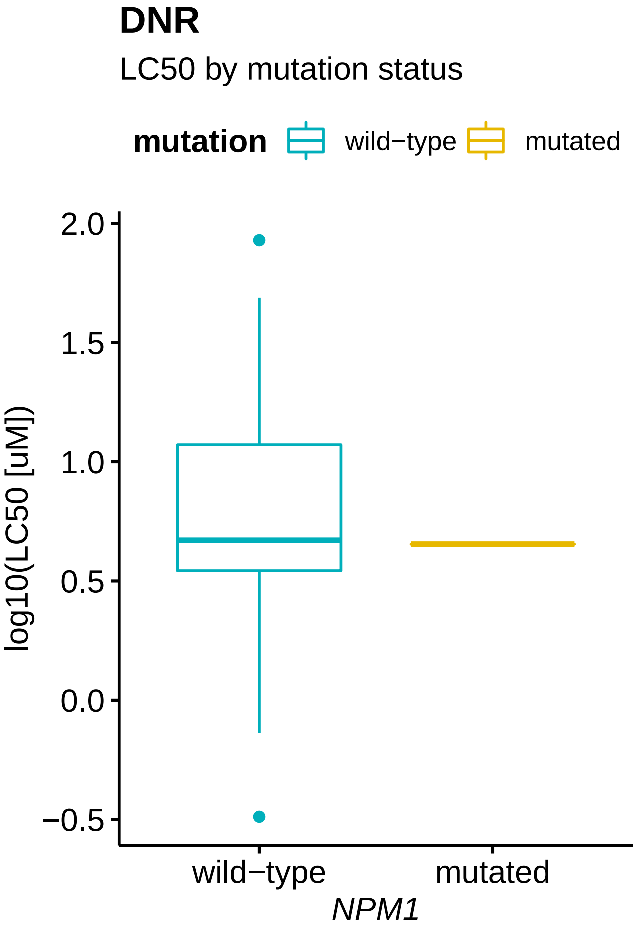
DNR
LC50 by mutation status
mutation
wild−type
mutated
2.0
1.5
1.0
0.5
0.0
−0.5
log10(LC50 [uM])
wild−type
mutated
NPM1

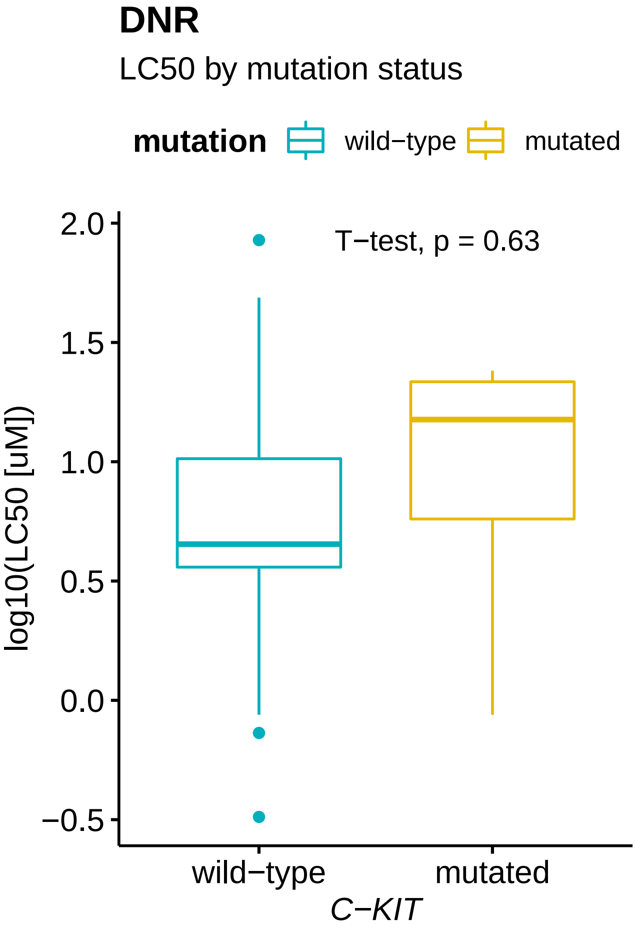
DNR
LC50 by mutation status
mutation
wild−type
mutated
T−test, p = 0.63
log10(LC50 [uM])
2.0
1.5
1.0
0.5
0.0
−0.5
wild−type
mutated
C−KIT

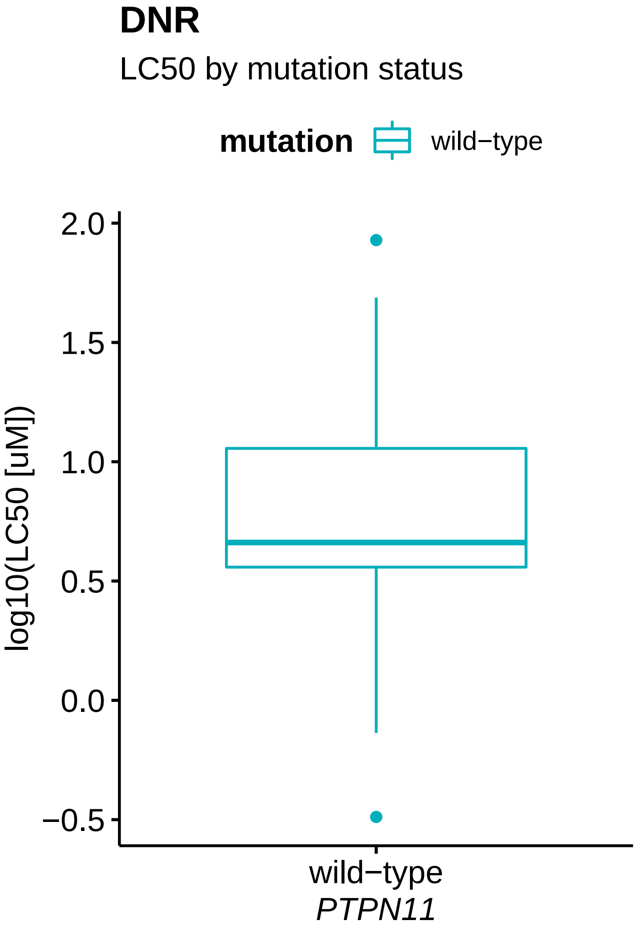
DNR
LC50 by mutation status
mutation
wild−type
log10(LC50 [uM])
2.0
1.5
1.0
0.5
0.0
−0.5
wild−type
PTPN11

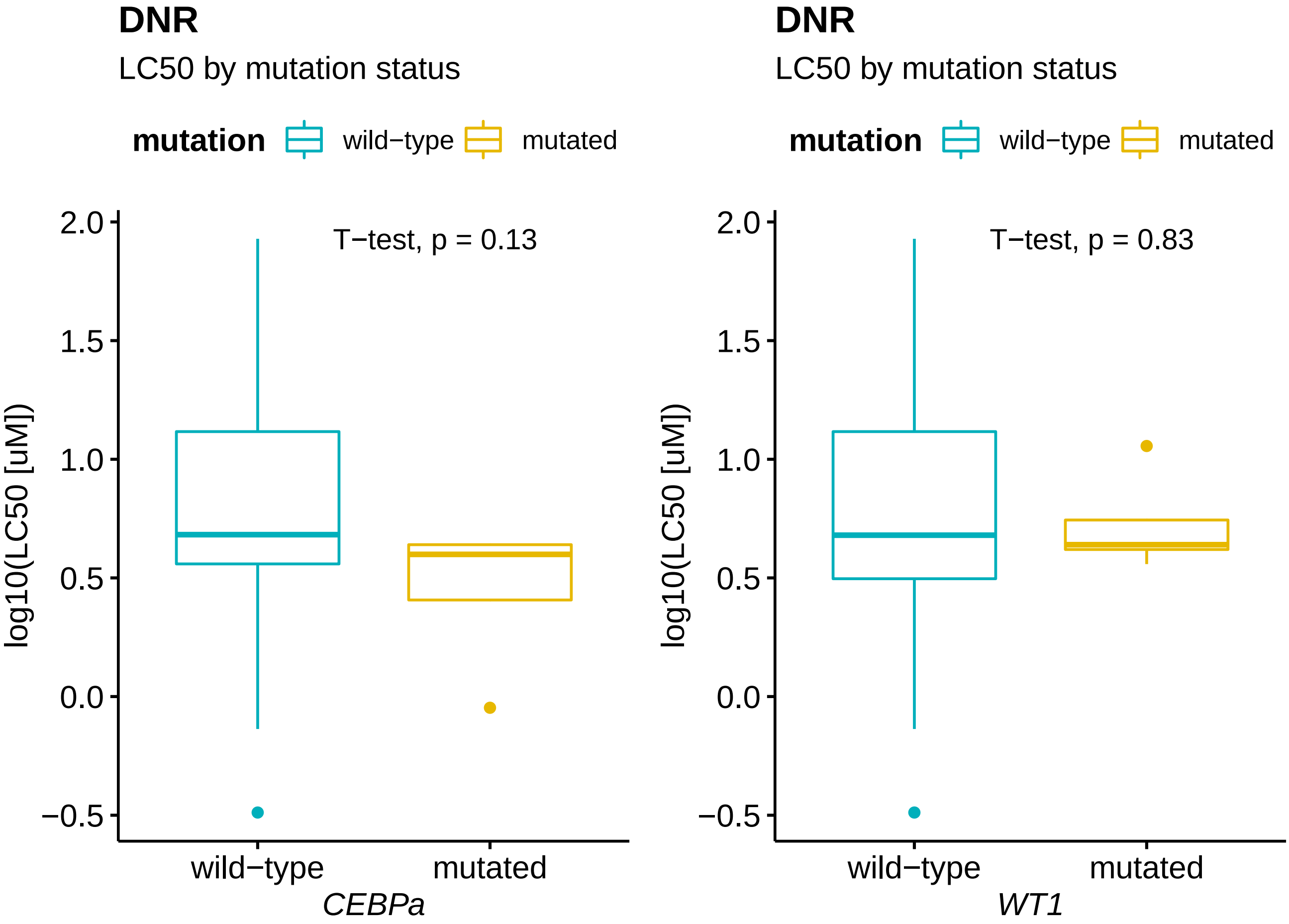
DNR
LC50 by mutation status
mutation
wild−type
mutated
T−test, p = 0.13
log10(LC50 [uM])
2.0
1.5
1.0
0.5
0.0
−0.5
wild−type
mutated
CEBPa
DNR
LC50 by mutation status
mutation
wild−type
mutated
T−test, p = 0.83
log10(LC50 [uM])
2.0
1.5
1.0
0.5
0.0
−0.5
wild−type
mutated
WT1

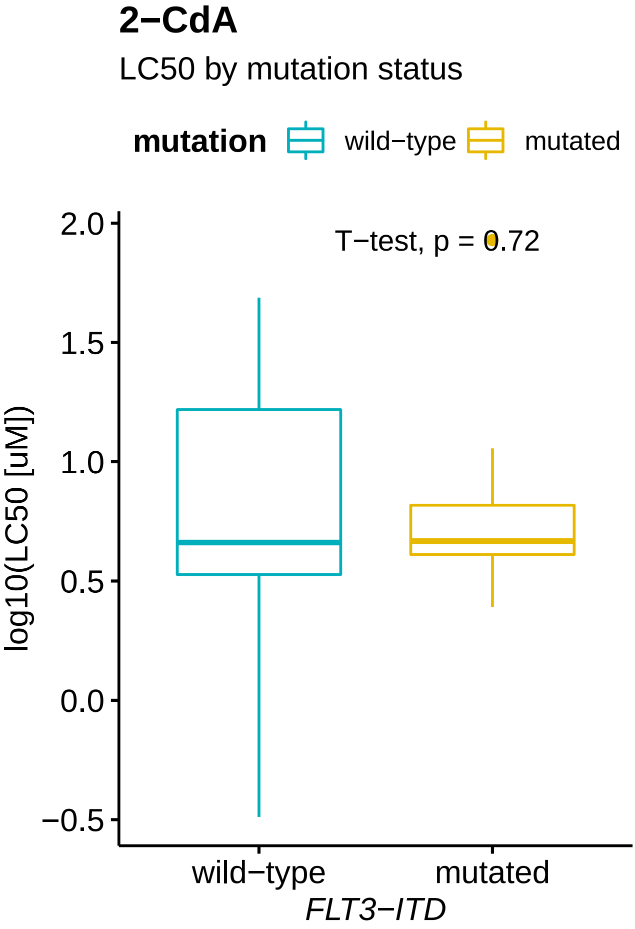
2−CdA
LC50 by mutation status
mutation
wild−type
mutated
T−test, p = 0.72
log10(LC50 [uM])
2.0
1.5
1.0
0.5
0.0
−0.5
wild−type
mutated
FLT3−ITD

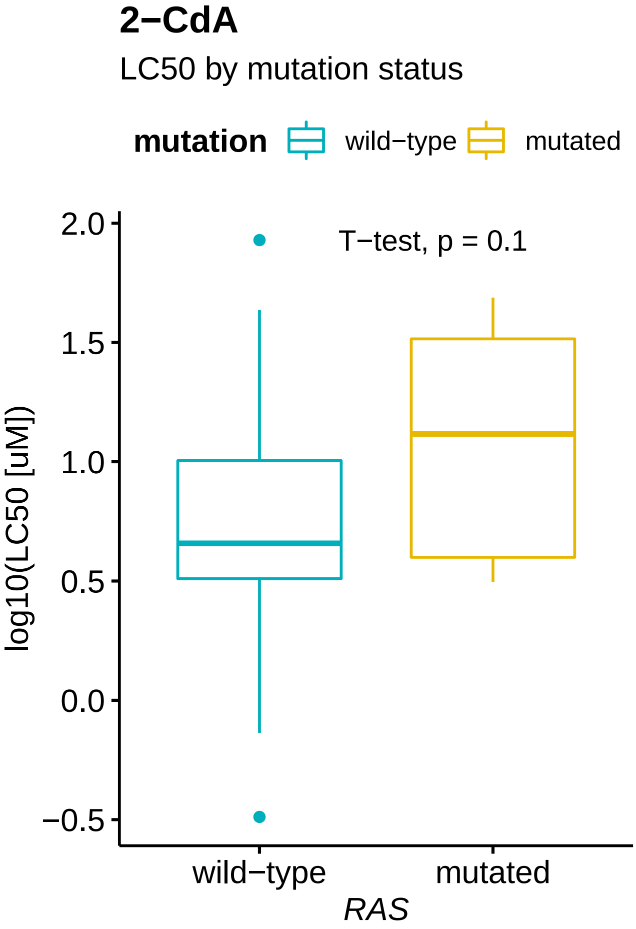
2−CdA
LC50 by mutation status
mutation
wild−type
mutated
T−test, p = 0.1
log10(LC50 [uM])
2.0
1.5
1.0
0.5
0.0
−0.5
wild−type
mutated
RAS

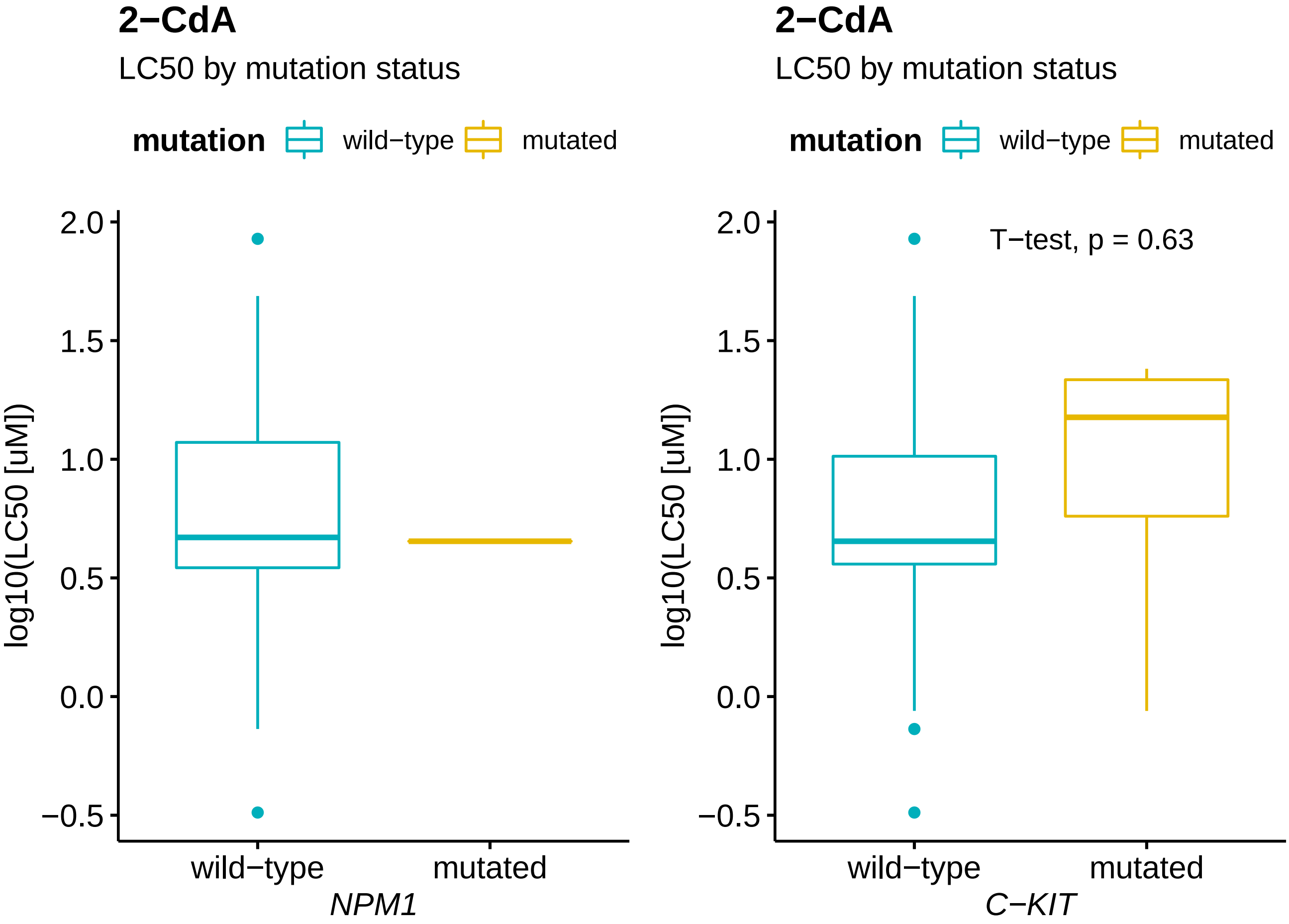
2−CdA
LC50 by mutation status
mutation
wild−type
mutated
log10(LC50 [uM])
2.0
1.5
1.0
0.5
0.0
−0.5
wild−type
mutated
NPM1
2−CdA
LC50 by mutation status
mutation
wild−type
mutated
T−test, p = 0.63
log10(LC50 [uM])
2.0
1.5
1.0
0.5
0.0
−0.5
wild−type
mutated
C−KIT

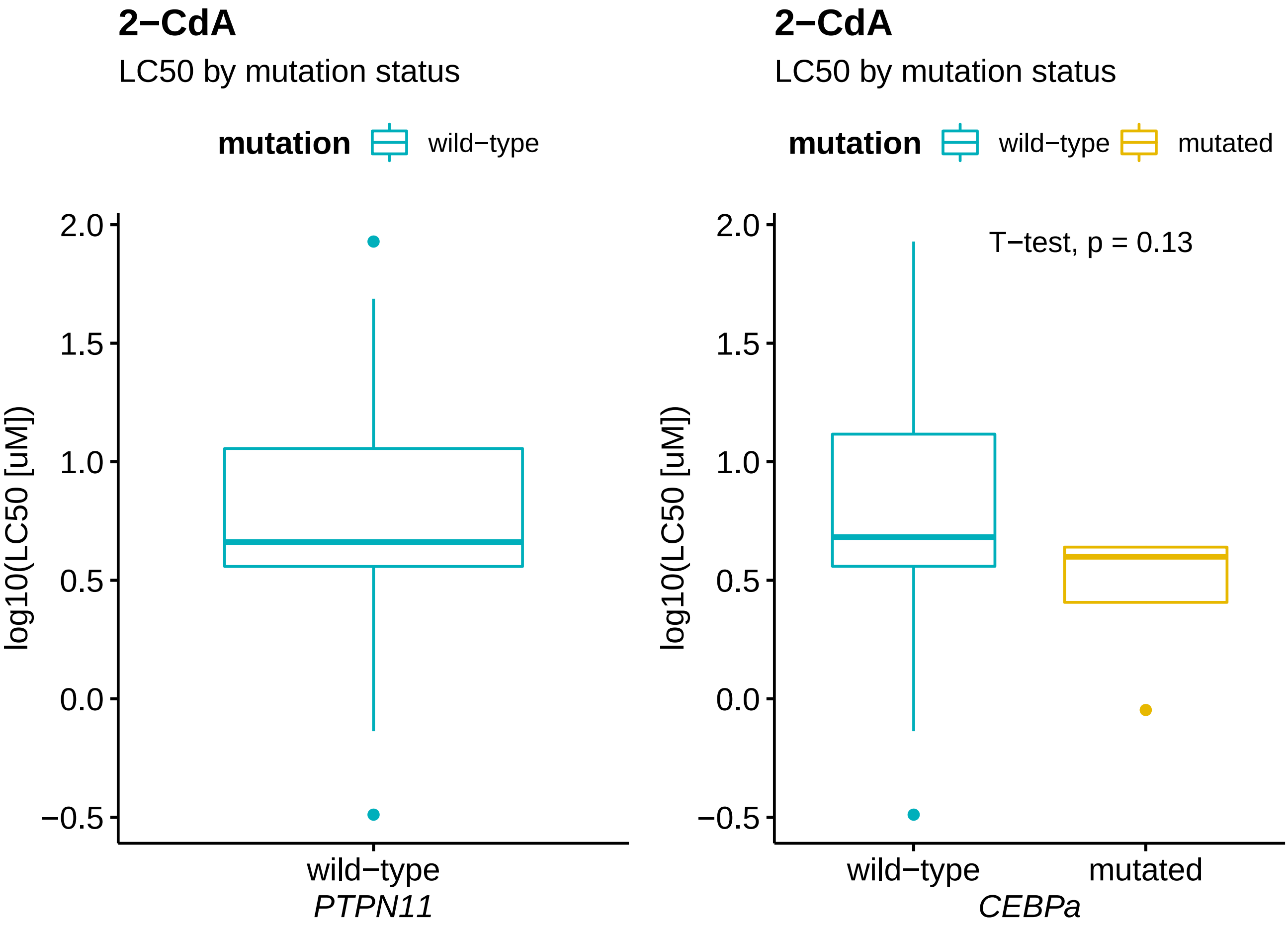
2−CdA
LC50 by mutation status
mutation
wild−type
log10(LC50 [uM])
2.0
1.5
1.0
0.5
0.0
−0.5
wild−type
PTPN11
2−CdA
LC50 by mutation status
mutation
wild−type
mutated
T−test, p = 0.13
log10(LC50 [uM])
2.0
1.5
1.0
0.5
0.0
−0.5
wild−type
mutated
CEBPa

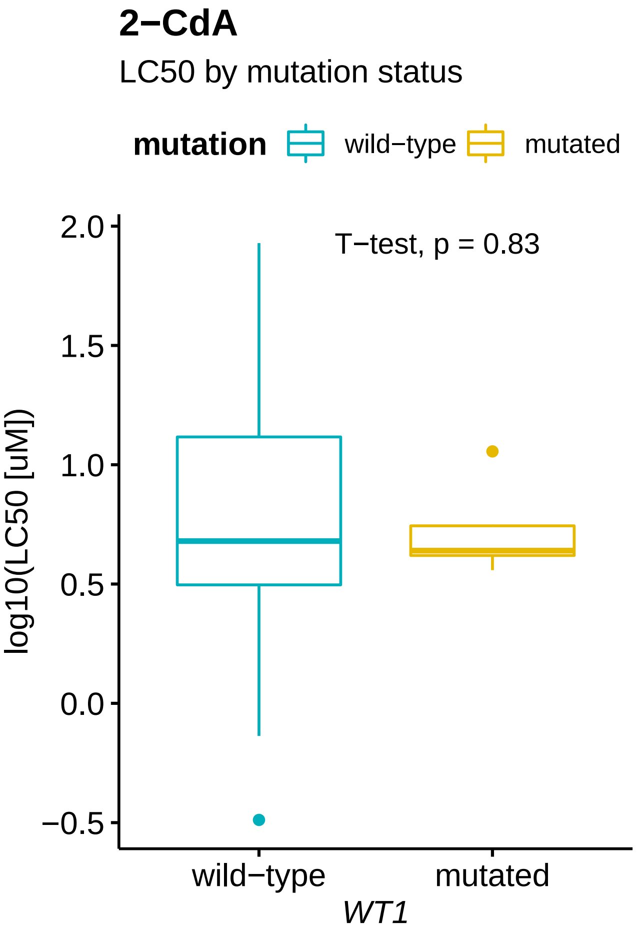
2−CdA
LC50 by mutation status
mutation
wild−type
mutated
T−test, p = 0.83
2.0
1.5
1.0
0.5
0.0
−0.5
log10(LC50 [uM])
wild−type
mutated
WT1

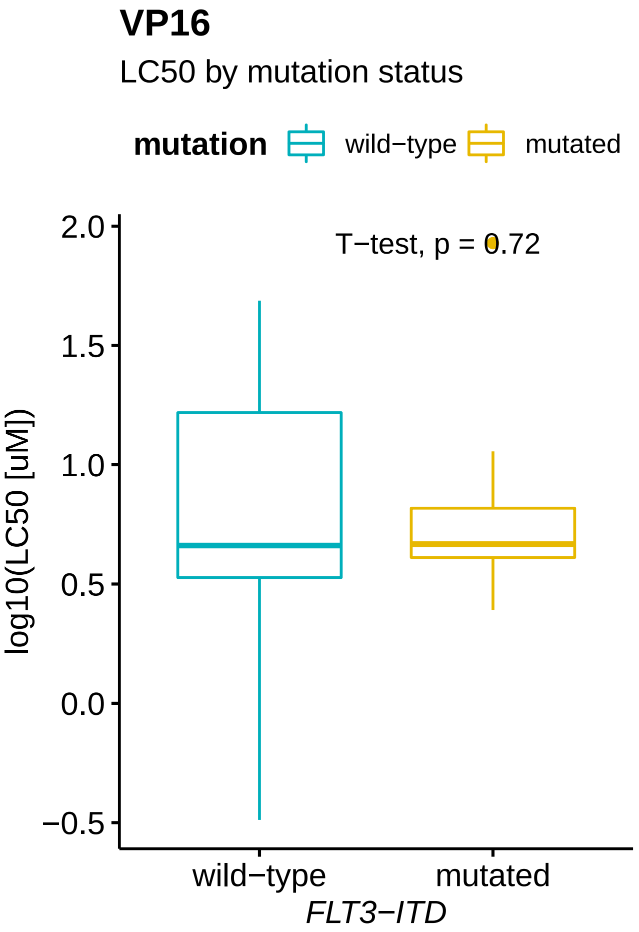
VP16
LC50 by mutation status
mutation
wild−type
mutated
T−test, p = 0.72
2.0
1.5
1.0
0.5
0.0
−0.5
log10(LC50 [uM])
wild−type
mutated
FLT3−ITD

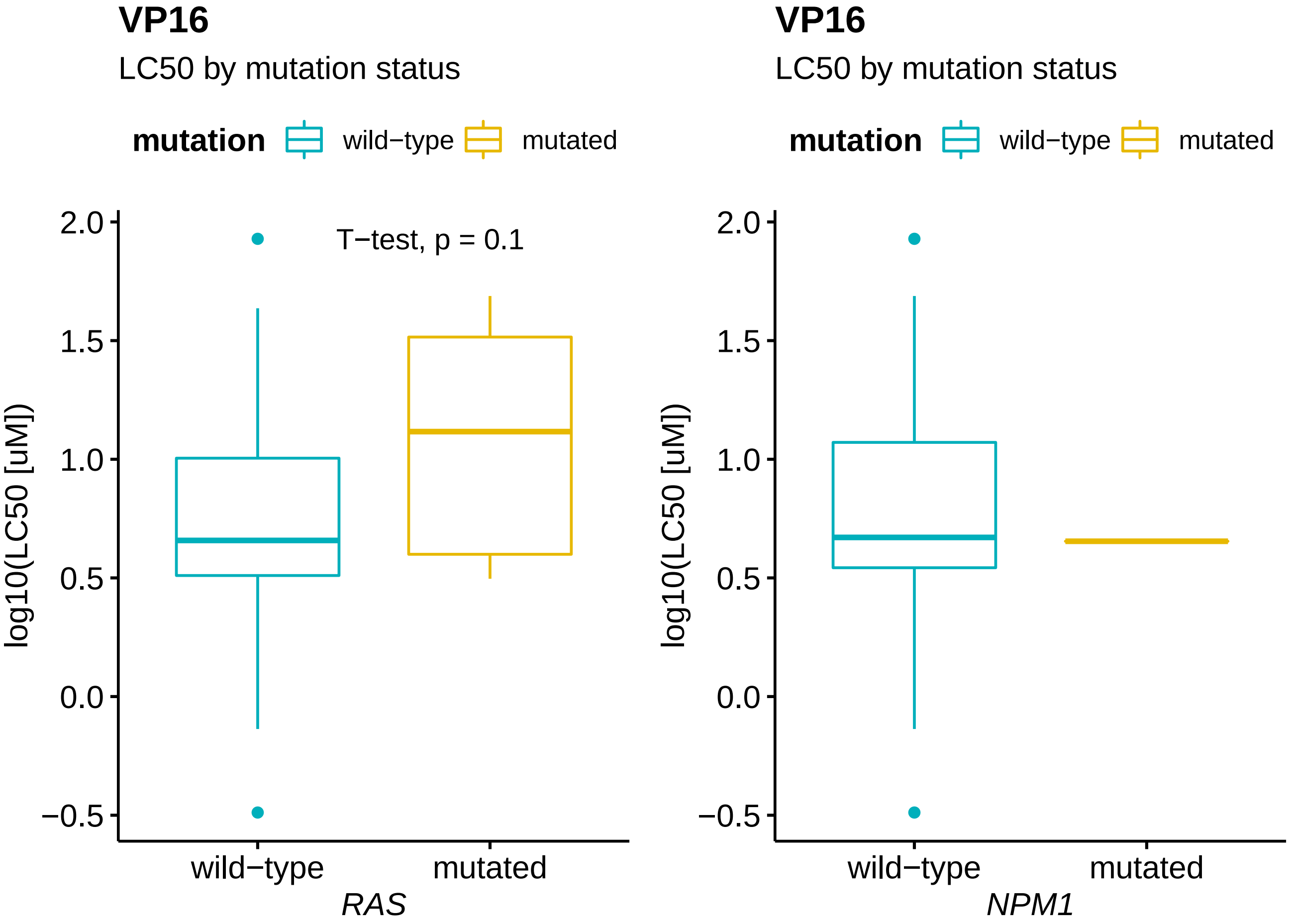
VP16
LC50 by mutation status
mutation
wild−type
mutated
T−test, p = 0.1
log10(LC50 [uM])
2.0
1.5
1.0
0.5
0.0
−0.5
wild−type
mutated
RAS
VP16
LC50 by mutation status
mutation
wild−type
mutated
log10(LC50 [uM])
2.0
1.5
1.0
0.5
0.0
−0.5
wild−type
mutated
NPM1

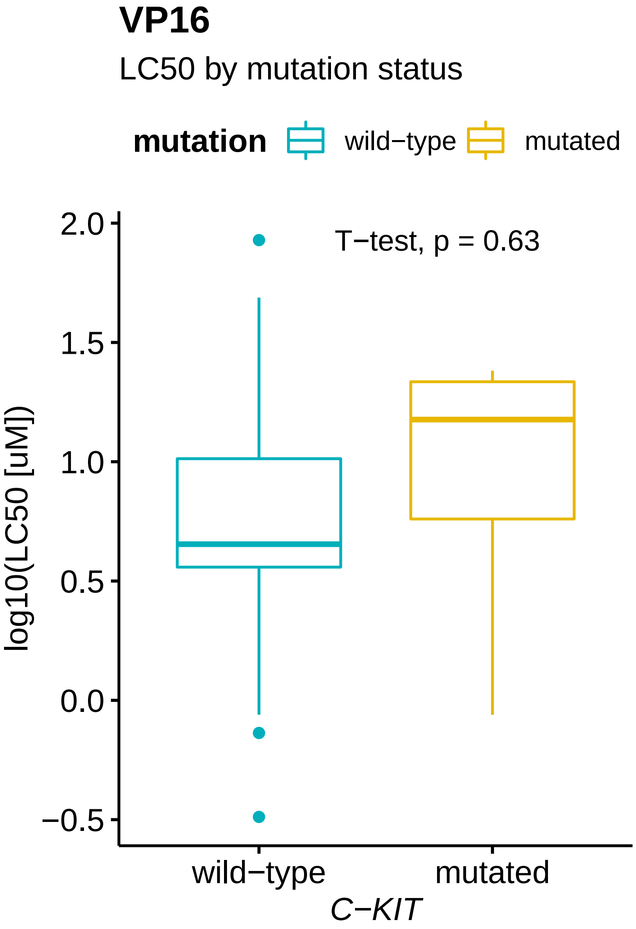
VP16
LC50 by mutation status
mutation
wild−type
mutated
T−test, p = 0.63
log10(LC50 [uM])
2.0
1.5
1.0
0.5
0.0
−0.5
wild−type
mutated
C−KIT

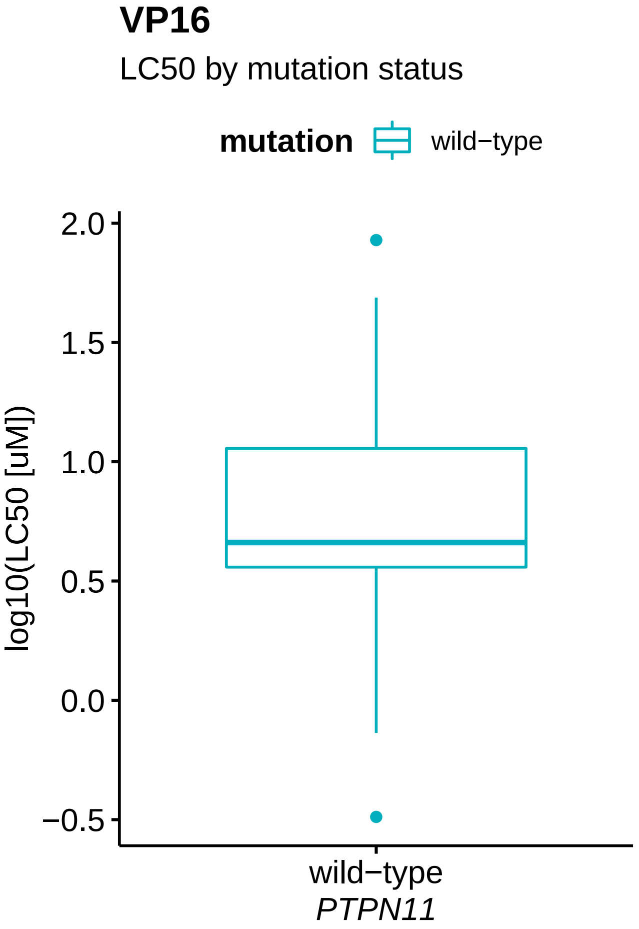
VP16
LC50 by mutation status
mutation
wild−type
log10(LC50 [uM])
2.0
1.5
1.0
0.5
0.0
−0.5
wild−type
PTPN11

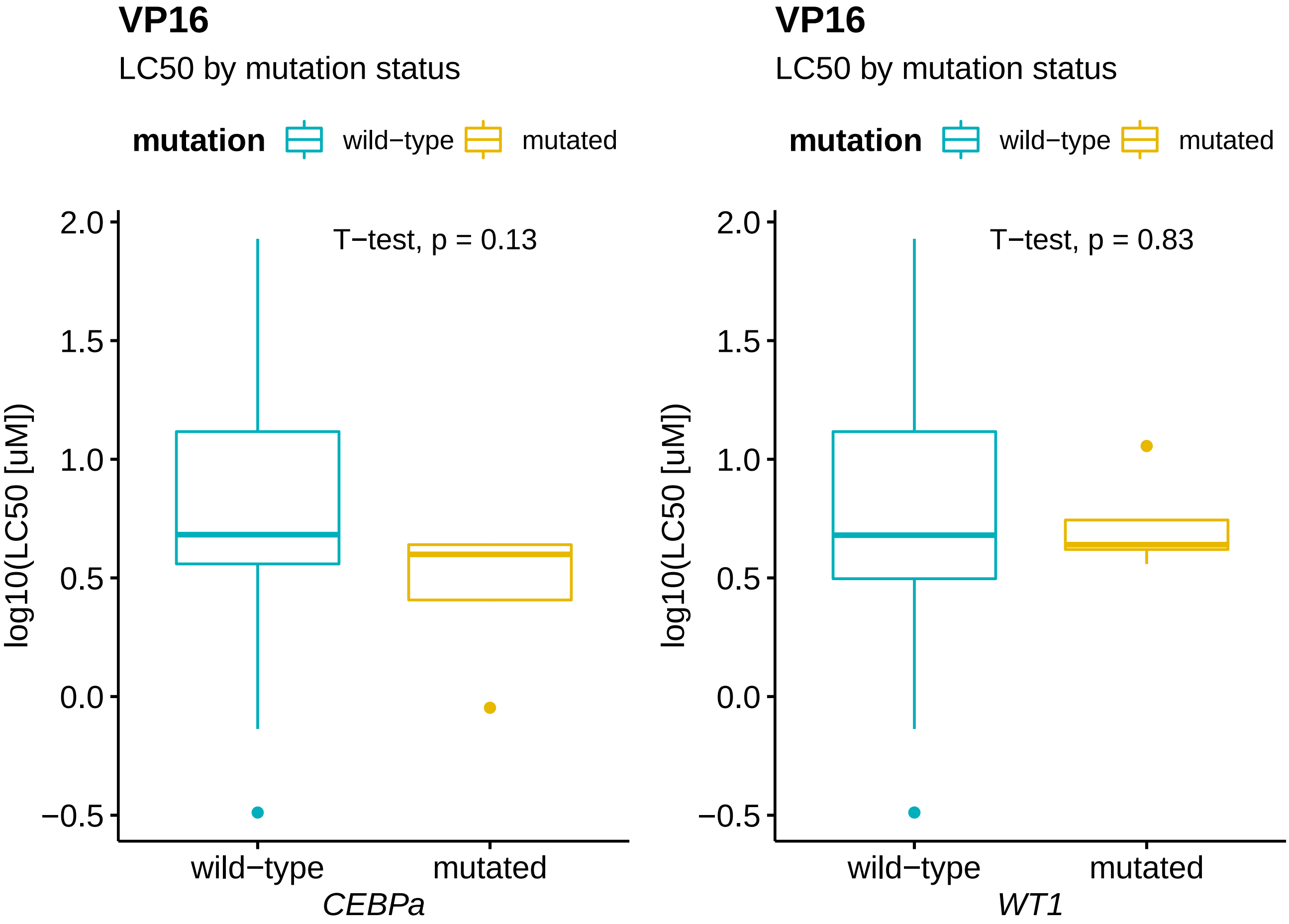
VP16
LC50 by mutation status
mutation
wild−type
mutated
T−test, p = 0.13
2.0
1.5
1.0
0.5
0.0
−0.5
log10(LC50 [uM])
wild−type
mutated
CEBPa
VP16
LC50 by mutation status
mutation
wild−type
mutated
T−test, p = 0.83
2.0
1.5
1.0
0.5
0.0
−0.5
log10(LC50 [uM])
wild−type
mutated
WT1

Supplementary Figure S2. Complete of Gene Ontology and Pathway analyses with genes associated with drug response towards (**A**) Ara C, (**B**) DNR, (**C**) 2-CdA and (**D**) VP16. (**E**): Significantly enriched genes involved in indicated biological processes associated with drug resistance towards VP16 are depicted in a gene interaction network.

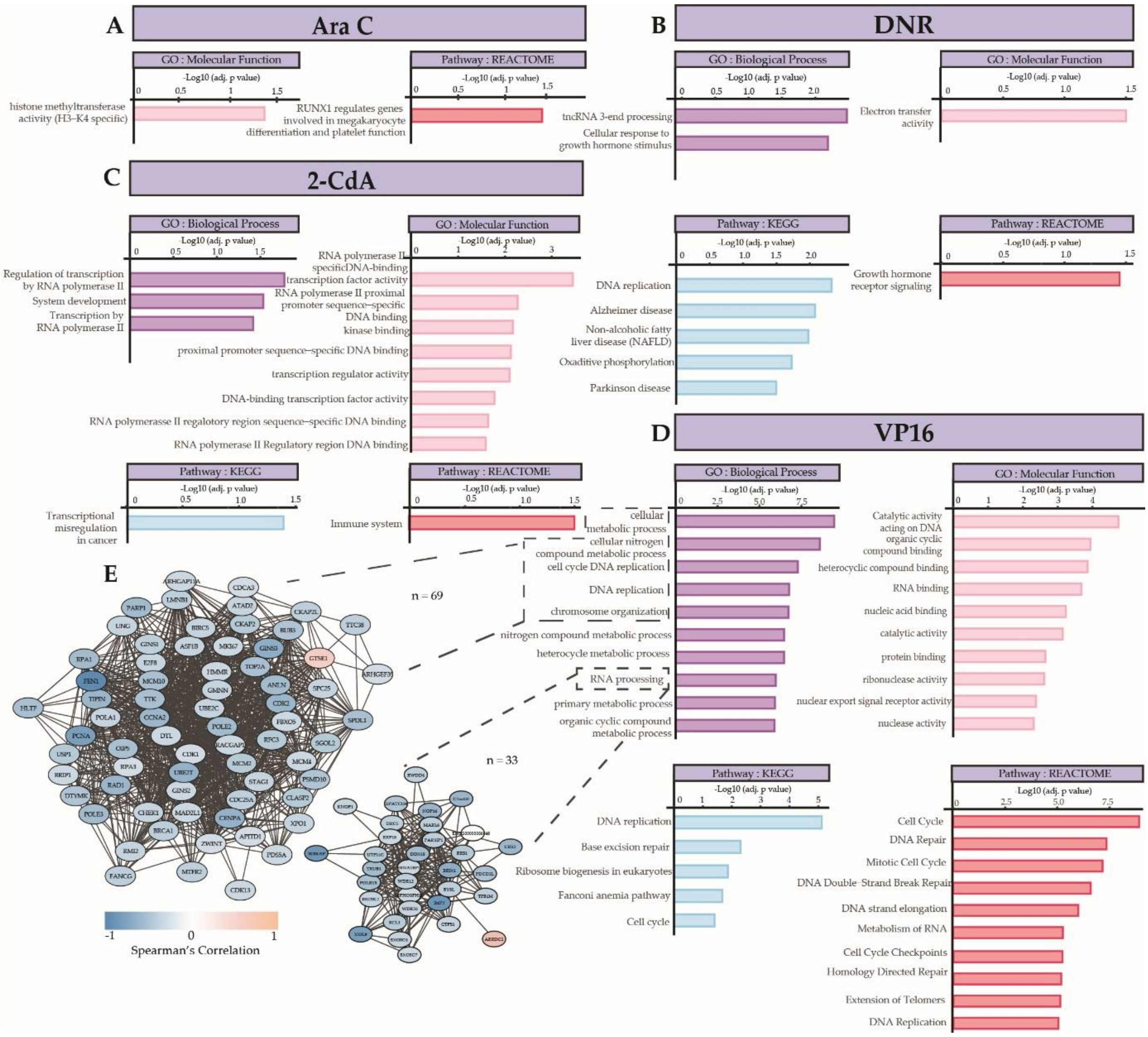

Supplement: Supplementary file 1 [file cancers-12-01247-s001.zip › Suppl zip/Supplementary Material Cancers.pdf]
